# Multidisciplinary, multicenter consensus for the care of patients affected with Sturge–Weber syndrome

**DOI:** 10.1186/s13023-024-03527-w

**Published:** 2025-01-16

**Authors:** May El Hachem, Andrea Diociaiuti, Angela Galeotti, Francesca Grussu, Elena Gusson, Alessandro Ferretti, Carlo Efisio Marras, Davide Vecchio, Simona Cappelletti, Mariasavina Severino, Carlo Gandolfo, Simone Reali, Rosa Longo, Carmen D’Amore, Lodovica Gariazzo, Federica Marraffa, Marta Luisa Ciofi Degli Atti, Maria Margherita Mancardi, and the Sturge-Weber Syndrome Multidisciplinary Group, Francesco Aristei, Alessandra Biolcati Rinaldi, Giacomo Brisca, Gaetano Cantalupo, Alessandro Consales, Luca De Palma, Matteo Federici, Elena Fontana, Thea Giacomini, Nicola Laffi, Laura Longaretti, Giorgio Marchini, Lino Nobili, Corrado Occella, Eleonora Pedrazzoli, Enrico Priolo, Giuseppe Kenneth Ricciardi, Erika Rigotti, Donatella Schena, Lorenzo Trevisiol, Urbano Urbani, Federico Vigevano

**Affiliations:** 1 Dermatology Unit, Genodermatosis Research Unit, Translational Paediatrics and Clinical Genetics Research Area, Bambino Gesù Children’s Hospital, IRCCS, Piazza San Onofrio, 4, 00165 Rome, Italy; 2 Dentistry Unit, Clinical Outcomes and Pathways Research UnitClinical Management and Technological Innovations Research Area, Bambino Gesù Children’s Hospital, IRCCS, Rome, Italy; 3 Plastic and Maxillofacial Surgery Unit, Technologic Innovations in Plastic Surgery Research Unit, Clinical Management and Technological Innovations Research Area, Bambino Gesù Children’s Hospital, IRCCS, Rome, Italy; 4 Ophthalmic Unit, Department of Surgical Odontostomatological Maternal and Child Sciences, Integrated University Hospital of Verona, Verona, Italy; 5 Neurology, Epilepsy and Movement Disorders Unit, Neurology and Neurosurgery Research Unit, Translational Paediatrics and Clinical Genetics Research Area, Bambino Gesù Children’s Hospital, IRCCS, Rome, Italy; 6 Neurosurgery Unit and Neurology and Neurosurgery Research Unit, Clinical Management and Technological Innovations Research Area, Bambino Gesù Children’s Hospital, IRCCS, Rome, Italy; 7 Rare Diseases and Medical Genetics Unit and Chromosomal Disorders and Dysmorphology Research Unit, Translational Paediatrics and Clinical Genetics Research Area, Bambino Gesù Children’s Hospital, IRCSS, Rome, Italy; 8 Neuroradiology Unit, IRCCS Istituto Giannina Gaslini, Genoa, Italy; 9 Functional and Interventional Neuroradiology Unit and Multimodal Imaging Research Unit, Clinical Management and Technological Innovations Research Area, Bambino Gesù Children’s Hospital IRCCS, Rome, Italy; 10 Department of Anaesthesia and Critical Care and Clinical Outcomes and Pathways Research UnitClinical Management and Technological Innovations Research Area, Bambino Gesù Children’s Hospital, IRCCS, Rome, Italy; 11 Epidemiology, Clinical Pathways and Clinical Risk Unit, Medical Direction; Clinical Outcomes and Pathways Research UnitClinical Management and Technological Innovations Research Area, Bambino Gesù Children’s Hospital, IRCCS, Rome, Italy; 12 Dermatology Department and Angioma Center, IRCCS Istituto Giannina Gaslini, Genoa, Italy; 13 Child Neuropsychiatry Unit, IRCCS Istituto Giannina Gaslini, Genoa, Italy; 14 Present Address: Pediatrics Unit, Neuroscience, Mental Health and Sense Organs (NESMOS) Department, Faculty of Medicine and Psychology, Sapienza University of Rome, Rome, Italy

**Keywords:** Sturge–Weber syndrome, Capillary malformation, Pulsed dye laser, Glaucoma, Epilepsy, Intellectual disability, Neuroimaging, Anaesthesia, Medical and surgical treatment, Psychosocial care

## Abstract

**Background:**

Sturge–Weber Syndrome (SWS) is a rare, sporadic neurocutaneous disorder affecting the skin, brain, and eyes, due to somatic activating mutations in *GNAQ* or, less commonly, *GNA11* gene. It is characterized by at least two of the following features: a facial capillary malformation, leptomeningeal vascular malformation, and ocular involvement. The spectrum of clinical manifestations includes headache, seizures, stroke-like events, intellectual disability, glaucoma, facial asymmetry, gingival hyperplasia, etc. An early diagnosis is crucial to guarantee an appropriate care, which is best performed in reference centres by multidisciplinary teams. The aim of this study was to develop a multidisciplinary expert consensus for diagnosis, treatment, and follow-up of all disease manifestations, according to the recommendations of the Italian Law on Rare Disease Care.

**Results:**

Through a Delphi consensus methodology, 28 recommendations have been developed concerning (i) dermatological SWS manifestations and related treatment timing and modalities, (ii) neurological referral, diagnosis, pharmacological treatment of neurological signs and symptoms, neurosurgical indications, neurocognitive evaluation and related treatment, psychosocial support and patient follow-up, (iii) diagnosis of ophthalmological manifestations, medical and surgical treatment, and follow-up, (iv) maxillofacial surgical treatment, (v) oral cavity assessment, care and follow-up, and (vi) primary care paediatrician/general practitioner involvement.

**Conclusions:**

The present consensus developed by a multidisciplinary group of experts from Italian reference centres comprises practical recommendations for SWS global management, including currently controversial issues. Specific statements for all disease aspects, from skin manifestations and neurological and ocular signs and symptoms to oral and maxillofacial care, are provided. They can be exploited to uniform clinical practice in reference centres, but also in other hospitals and outpatient settings. Though this consensus has been developed taking primarily into account the Italian National Health System organization and rules on rare disorders, it could be translated also to other countries.

**Supplementary Information:**

The online version contains supplementary material available at 10.1186/s13023-024-03527-w.

## Background

Sturge–Weber Syndrome (SWS) is a sporadic neurocutaneous disease involving the skin, brain, and eyes. It is due to somatic activating mutations in guanine nucleotide-binding protein alpha-q or alpha-11 subunit encoded by *GNAQ* and *GNA11* genes located on chromosome 9q21 and 19p13, respectively [1, 2]. It occurs in 1 in 20,000 to 1 in 50,000 live births and is characterized by at least two of the following features: cutaneous capillary malformation (port-wine stain), cerebral vascular malformation, and ocular involvement [1, 2]. Clinical course is unpredictable. Patient management is aimed to perform early diagnosis and to provide appropriate treatment.

Capillary malformations (CMs) are low-flow vascular malformations that affect up to 0.5% of newborns [1]. They are present since birth as pink to red, persistent, and well-demarcated patches. Histologically, they are characterized by clusters of superficial capillaries abnormally increased in size and number. In the last decade, several authors described the facial localization and distribution of CM that can predict SWS, with implications for the screening of children who should be investigated for neurological and ocular involvement [1–3]. CM can also affect the oral mucosa with subsequent gingival and dental manifestations. Moreover, pyogenic granulomas can develop on the skin and mucosal CMs and may bleed spontaneously. Over time, CMs may become darker with underlying soft tissue and skeletal hypertrophy occurring in 65% of cases by the fifth decade of life [4]. Facial and gingival overgrowth can cause facial asymmetry as well as bleeding from gums and difficulties to chew, requiring plastic surgeon and dentist/oral surgeon care [5].

The neurological phenotype resulting from cerebral vascular malformations is extremely variable among patients and at different ages in the same patient. It includes seizures, stroke-like episodes, headache, hemiparesis, visual field deficits, intellectual disorders, and behavioral issues [1, 6]. The natural history of SWS neurological manifestations is poorly delineated, and pathophysiology is not yet clear [7]. Few prognostic indicators may predict the neurological course, in particular early seizure onset, high seizure frequency and bilateral involvement are related to worse cognitive outcomes [2]. Ocular involvement may include episcleral or choroidal vascular malformation, iris heterochromia, and most frequently glaucoma, affecting 30–70% of patients [8–10].

Overall, SWS manifestations markedly affect the quality of life of patients and their families. In addition to consequences of neurological and ocular symptoms and signs, patients experience high level of anxiety and decreased self-esteem with frequent difficulties in relationships due to cutaneous CMs. This impact increases with age and improves significantly when CM is treated [11].

To date, recommendations for the management and treatment of SWS have been developed in the U.S. In 2018, the Sturge–Weber Foundation published a review regarding the pathogenesis, clinical manifestations, and treatment options for SWS [7]. In 2021, two U.S. consensus statements were developed to provide practical recommendations for the management of skin CMs, neurological, and ocular manifestations of SWS [12, 13].

Our aim was to develop multidisciplinary practical consensus recommendations for the diagnosis, treatment, and follow-up of all cutaneous and extracutaneous disease manifestations, according to the Italian Law on Rare Disease Care [14]. A multidisciplinary expert group (dermatologists, anesthesiologists, ophthalmologists, child neuropsychiatrist, neurologists, neurosurgeons, neuroradiologists, dentists, plastic surgeons, pediatricians, and psychologists) developed these recommendations. Experts were from three Italian reference centers, whose representatives are members of the Scientific Committee of the Italian Sturge–Weber Patient Association (https://sturgeweberitakia.org/). In addition, all participant institutions are full members of a European Reference Network (ERN) for rare and complex epilepsies, EpiCARE (https://epicare.eu/), which is related to SWS care. Finally, Bambino Gesù Children’s Hospital is also member of three additional ERNs implicated in SWS management: ERN-Skin for rare and undiagnosed skin disorders (https://ern-skin.eu/), the VASCA group of VASCERN for rare vascular anomalies (https://vascern.eu/groupe/vascular-anomalies/), and ERN-ITHACA for rare malformation syndromes, intellectual and other neurodevelopmental disorders (https://ern-ithaca.eu/).

## Methods

### Design

For this study, we used the Delphi consensus methodology. This procedure is a well-established instrument for reaching consensus between a panel of experts for research questions that cannot be answered with empirical evidence and complete certainty [15]. It is an iterative technique based on the scoring of a series of structured statements that are revised, fed back to the participants, and repeated in multiple rounds, in increasing detail, until consensus has been reached [16].

### Participants

National experts of different specialties were involved in this study during the period March 2022- March 2023. In particular, the writing group was composed of 4 dermatologists, 2 neurologists, 1 neurosurgeon, 2 neuroradiologists, 2 ophthalmologists, 1 dentist, 1 plastic surgeon, 1 anesthesiologist, 1 pediatrician, and 1 psychologist. Their selection was based on the expertise in the management and treatment of SWS. The three Italian Hospitals (Bambino Gesù Children’s Hospital, Rome; IRCCS Istituto Giannina Gaslini, Genova; University Hospital of Verona) were involved based on their membership to the Scientific Committee of Italian Sturge–Weber Patient Association. Within the Delphi process, votes of all experts including the members of the SWS multidisciplinary group are weighted equally. SWS multidisciplinary group members participating to the voting process comprised: 2 dermatologists, 3 neurologists, 1 neuroradiologist, 3 child neuropsychiatrists, 1 neurosurgeon, 3 ophthalmologists, 2 maxillofacial surgeons, 2 dentists, 1 anesthesiologist, 2 pediatricians, and 1 psychologist. Moreover, one patient representative was a non-voting member of the group. Finally, two epidemiologists participated to the study for methodological support and data analysis.

### Elaboration of recommendations

Six major topics were identified (dermatology, neurology, ophthalmology, plastic surgery, dentistry, and pediatrics), and the writing group members assigned to the different topics. For each topic, experts generated recommendations based on both their experience in the management and treatment of SWS, and extensive literature review of articles published from 1990 to 2022. Statements were shared among the experts and voted.

### Data collection

An online Delphi procedure was performed over two rounds. All statements were voted anonymously by each participant using Google Forms. In each round, voting members were e-mailed a unique link to the voting platform. The results of the voting for statement were reported to participants at the end of each round. The results were presented anonymously by area of expertise.

### First and second round

All recommendations on management and treatment of SWS by area of expertise were presented to the SWS multidisciplinary group during the first round, and it was asked to participants to rate them on a 5-point Likert scale (1, strongly disagree; 2, disagree; 3, neutral; 4, agree; 5, strongly agree). Recommendations rated ≥ 3 by at least 79% of experts were included in the consensus; those rated ≤ 2 by more than 21% of experts were discussed by all the experts and eventually rephrased. Modified recommendations were presented and scored by all the experts in the second round for agreement (i.e. rating ≥ 3 by at least 79% of experts).

## Results

Thirty-three experts participated to the two rounds of discussion and voted 28 statements and relative recommendations, out of which 17 (60.7%) were included in the consensus at first round. Eleven (39.3%) statements were rephrased and discussed again during the second round. After the second round, all the proposed statements and recommendations were included in the consensus (see Additional File 1, Table S1).

### Dermatological recommendations

#### Which distribution of the capillary malformation (CM) on the face should lead to the suspicion of Sturge–Weber syndrome (SWS)?

CMs of the face involving the frontal region, bordered inferiorly by a line joining the outer canthus of the eye to the upper portion of the ear and encompassing the upper eyelid, as well as CMs of the middle frontonasal region should suggest suspicion of SWS.

**Fig. 1:**
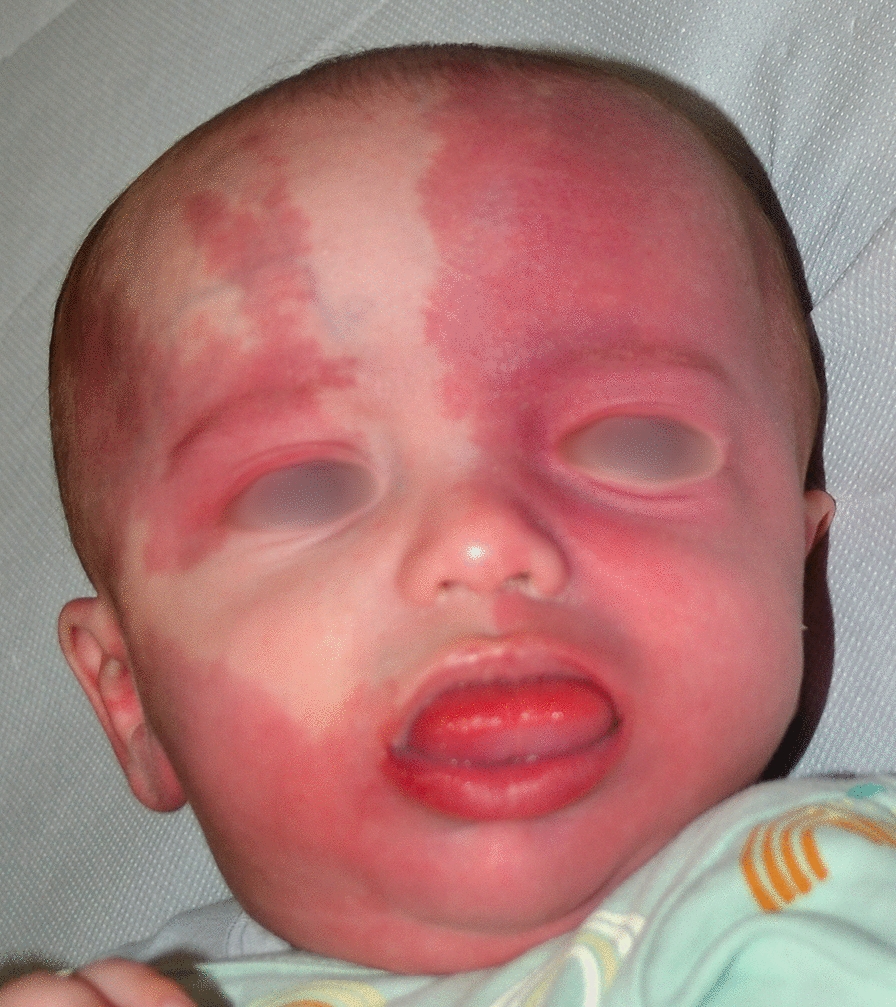
Bilateral capillary malformation involving most of the face in an infant with Sturge–Weber syndrome, who developed epilepsy during the first months of life

CM in SWS is mostly localized to the frontal and/or fronto-temporal lateral region, it may be mono- or bilateral and, less frequently, it is distributed on the medial frontonasal prominence [1, 4, 6, 12] (Fig. 1). A consensus statement by Sabeti et al., comprising a systematic review of the literature from 2008 to 2018, identified the distribution of CMs of the head with increased risk for association with leptomeningeal and/or ocular anomalies [12]. In 2014, Waelchli et al. reported that a CM distributed to the fronto-temporal region is the strongest predictor of risk to develop SWS [2]. In particular, a CM localized to any part of the forehead, delineated at its lower edge by a line joining the outer canthus of the eye to the upper part of the ear, and including the upper eyelid and the frontonasal prominence of the midline, should be considered the strongest predictor of risk of SWS. Indeed, 83 out of 103 children (80.6%) with a similar distribution of CM were affected with SWS [2]. Dutkiewicz et al. in a multicenter prospective study reported two phenotypes significantly associated with SWS: complete hemifacial and median involvement [17]. The same distribution of CMs as an indication to screen for SWS was reported by Zallmann et al. [18]. As the anatomical areas that originate from the frontonasal prominence develop their vasculature from the forebrain and anterior midbrain, CMs distributed in these regions should suggest the risk of SWS, overcoming the long-standing belief of a "trigeminal nerve" etiology [19]. Of note, SWS CM affecting the frontonasal prominence should be differentiated from the much more common nevus simplex (also known as salmon patch) which may be present at multiple sites (upper eyelids, nape, scalp vertex and/or the back), has poorly defined borders, and progressively fades starting from the first weeks of life.

#### What is the most appropriate age to start pulsed dye laser (PDL) treatment?

The most appropriate age to start PDL treatment for CM is early childhood. Early treatment improves the effectiveness of therapy, and quality of life (QoL) of the patient and parents.

It is currently correct to inform parents of the two approaches, in sedation or with anesthetic cream, and their related risks and benefits. The use of sedation, reducing the frequency of sessions, is recommended to avoid physical and psychological suffering and its consequences. In children, sedation is always necessary for treatment of the orbital region.

Photocoagulation by PDL is the first choice for the treatment of CMs [12, 20]. It acts by selective photothermolysis of hemoglobin, and the target are superficial vascular structures: dilated capillaries and post-capillary venules of the reticular and papillary dermis localized at an average depth of 1.2–1.5 mm [21]. PDL is considered the most effective and safe treatment of CM in patients of all ages [22]. The main goal of early treatment is to minimize the stigma associated with CM, ensuring healthy psychosocial development as well as a better QoL for both patient and parents [12, 23–26]. The efficacy of the treatment is greatest when performed in early childhood and on small lesions ≤ 20 cm^2^ [27–31]. Based on expert observations and limited studies, treatment of CM at an early age, particularly in the first year of life, leads to better results [12, 32]. At birth, the skin is approximately 40%-60% thinner than that of the adult, with less melanin and fewer hair follicles, allowing better laser penetration and less scattering [27]. In the newborn, CM presents as a proportionately smaller superficial pink patch, and, due to the above physiologic and anatomic features, it is considered the optimal target of PDL [20]. Most CMs evolve, with a progressive tendency to darken, thicken and increase in size, features that reduce the efficacy of dye laser. Finally, some authors suggest that PDL treatment may prevent the onset of proliferative nodules, pyogenic granulomas, and soft tissue hypertrophy, which usually appear over the years [26, 27].

Multiple treatments are needed, spaced 4–6 weeks, in order to achieve optimal results. Several studies showed that early intervention improves the outcome [26, 33, 34]. In addition, early treatment may reduce the number of analgesia/sedation procedures required, and may decrease the psychological distress of the patient, due to the lesion fading before the age of consciousness.

Recent studies report efficacy and safety of PDL treatment for CM without local or general anesthesia in newborns and infants (5–355 days) with a mean of 9.8 treatments. Patients were immobilized and eyes protected by placing stainless-steel corneal shields. Good clearance without scarring or permanent pigmentary changes has been described [32, 33]. However, no studies were performed to evaluate the impact of pain memory in the long-term.

#### Is PDL without sedation recommended in the first year of life?

Exposing the infant to pain is inadvisable. In contrast, the use of sedation/general anesthesia under the age of one year for PDL treatment of CM is a practicable and preferred strategy. Sedation should be considered as part of a course of care shared by a multidisciplinary team and individualized with respect to the patient's clinical condition and needs.

Infants younger than 18 months of age have greater pain perception than at later ages. In fact, pain regulatory pathways are not developed until 12–18 months of age, while nociceptive transmission pathways are fully functional [35]. The central nervous system in the neonatal age is characterized by marked plasticity, and an increased risk of neurobehavioral alterations from exposure to painful stimuli, including short- and long-term physical and psychological disability, impaired cognitive development, and altered somatosensory perception [35]. Procedural pain is an important cause of stress and anxiety for the child, particularly for children with chronic diseases. Therefore, pain should be monitored and measured with appropriate scales according to the developmental level [36]. In addition to perceiving more intense pain than adults for the same stimulus, children develop a "pain memory" from the neonatal age [37].

In the pre-procedural period, it is crucial to inform parents about the pain prevention/control program that will be used. The child should present to the procedure pain-free, going through anesthesia during PDL treatment and ending with postoperative analgesic therapy, if required.

In 2007, the Food and Drug Administration Anesthetic and Life Support Drugs Advisory Committee released recommendations aimed at containing neurotoxic risk from anesthetic agents. The advice came from pre-clinical studies and ongoing trials to evaluate possible long-term adverse effects. These statements, however, are not yet confirmed by clear evidence [38, 39]. Meanwhile, recent randomized controlled trials have shown no difference in neurocognitive development between subjects undergoing general and locoregional-conscious anesthesia in the neonatal and infant periods [40], identifying severe critical cardiovascular and respiratory events as the main cause of medium- and long-term sequelae. The degree of training and experience of the medical team along with the adoption of standardized guidelines for perioperative management are the instruments to avoid these complications [41, 42].

In the common clinical practice, the above data have been transposed as an indication to postpone the course of PDL treatment under sedation or general anesthesia after the age of 6 months, keeping the possibility of performing early procedures under topical anesthesia. The latter is an option that has proven feasible with better results in the infant age group (< 1 year), also in view of smaller skin area involved and the greater ease in keeping the patient still while guaranteeing his/her safety. However, it is important to consider the effect generated by exposure to repeated painful stimuli over time, that may result in short- and long-term negative consequences on behavior, closed attitudes toward care, stress and dissatisfaction in the family environment [43]. Therefore, the decision to resort to sedation/general anesthesia techniques under one year of age is a viable strategy, that should be carefully considered as part of a care pathway shared by a dedicated multidisciplinary team, and individualized with respect to the clinical conditions and patient needs.

#### Which is the recommended sedation modality to perform PDL?

Sedation of a patient with SWS should be chosen based on a careful medical history and a complete clinical assessment. In the absence of neurological symptoms in the past 3 months, the anesthesiologic approach is not different from that adopted in the healthy pediatric population of comparable age. In patients with neurological symptoms that cannot be controlled by therapy, sedation is recommended with a post-procedural observation of at least 24 h. The combination of topical anesthetic cream with airway administration of sedative medications provides the best conditions for treatment of uncooperative patients. A recent (within the last 3 months) electroencephalogram (EEG) tracing in patients on anticonvulsant therapy is recommended.

The sedation treatment regimen for patients with SWS should be based on a careful clinical history, physical examination and evaluation of blood and radiological investigations [44].

Relevant aspects to be considered are: (i) maintenance of airway patency (sometimes site of vascular malformation), (ii) maintenance of the capacity for spontaneous ventilation, (iii) maintenance of normal values of intracranial pressure, intraocular pressure, systemic arterial pressure, in order to avoid the occurrence of stroke-like events or acute ocular damage, and (iv) pharmacological prevention of stimulation of potential epileptogenic foci, through the choice of drugs without proconvulsant action.

A pharmacological strategy with the association of topical (lidocaine/prilocaine cream) and i.v. anesthesia such as midazolam/dexmedetomidine finds rational use. Local anesthesia allows local sensory blockade of the area to be treated with PDL. Midazolam is a short half-life benzodiazepine with sedative action that produces anxiolysis and memory loss of any discomfort or undesirable effects that may occur during the procedure. It may be administered orally or intranasal. Dexmedetomidine is a sedative sympatholytic analgesic drug acting also on the anxiety component. Moreover, there are several studies indicating dexmedetomidine as the only agent with a demonstrated neuroprotective effect, capable to reduce the neuroinflammatory processes underlying the mechanisms of cerebral neuroapoptosis observed in children [45]. Both molecules can be administered alternatively, or in combination in a single aqueous solution. They do not exhibit pro-convulsant action due to their pharmacological properties. It is known that pharmacological treatment of epilepsy has a variable and frequently unpredictable efficacy. Some patients have long seizure-free periods even in the absence of pharmacological therapy, while others may have frequent and prolonged seizure despite ongoing treatment [46]. A history of clinical stability, with absence of seizures for at least the last 3 months prior to treatment, whenever possible, is the most significant element in assessing a patient's clinical suitability for the procedure under outpatient sedation, even in patients requiring repeated treatment sessions. An interval between treatment sessions for efficacy of PDL treatment and safety could range between 2 weeks and 3 months [12].

### Neurological recommendations

#### Are neurological visit and electroencephalogram (EEG) recommended for children with clinical suspicion of SWS?

Any child with facial CM at high-risk for SWS should be referred to a specialist expert in pediatric neurology for a baseline evaluation (clinical history and neurological examination) and EEG (during sleep, better if 30 min, at least 8 channels), preferably during the first three months of life.

Early recognition of patients at high risk for epilepsy is crucial since ongoing seizures in infancy can adversely affect neurodevelopment [47]. Under the assumption that early detection and prompt treatment can result in better neurological outcomes, added to the possibility of false negative early magnetic resonance imaging [18), a neurological visit with EEG is recommended in infants with facial CM at high risk for SWS, even in the absence of neurological signs/symptoms. In fact, the neurological evaluation can offer counselling about early seizure recognition, triggering factors and emergency treatment plan [7]. It is suggested to perform neurological examination during the first months of life since in most cases seizures start during the first year [6, 13]. The EEG, although not diagnostic, can be useful since it can detect amplitude asymmetry, focal slowing, epileptiform abnormalities, or even subclinical seizures. The recording, at least 8 channel scalp EEG, should last 30 min, and it is preferable to obtain a sleep tracing, with polygraphic channels, to reduce movement artifacts and increase the possibility to recognize epileptiform abnormalities. In asymptomatic patients who show epileptiform abnormalities or marked asymmetry in EEG during early evaluations or follow-ups, we recommend to reiterate parents’ education for subtle seizures recognition and management and to personalize timing of follow-up. In these cases, it appears reasonable to wait till 12 months of age to perform MRI, in order to minimize possibility of false negatives and avoid repeated anesthesia and gadolinium injections.

#### Are follow-up neurological visits and EEGs indicated for patients with confirmed SWS?

Longitudinal follow-up with clinical neurological evaluations and EEGs is indicated for infants with confirmed SWS, preferably every 6 months during the first 2 years of life for parental education and monitoring of neurologic red flags and seizures.

Children with SWS are at high risk for seizures and neurocognitive deficits [48], and clear prognostic biomarkers are still lacking. In this panorama, the EEG represents a valuable non-invasive tool, since rhythm and voltage asymmetry and/or epileptiform abnormalities on EEG might be a useful marker to identify patients with SWS at risk of developing epilepsy [6, 49–52]. Moreover, the EEG in SWS frequently evolves, becoming more abnormal with increased epileptiform activity over time [52, 53].

One of the priorities in the follow-up of these patients is a continuous surveillance by a child neurologist/neuropsychiatrist, in order to detect subtle seizures and neurologic red flags, that include the onset of visual field deficits, early hand preference, automatisms, nystagmoid movements, and developmental delay or plateau[18]. The timing of further examinations beyond the age of 2 years should be decided by the specialist depending on the clinical picture and risks.

#### Is brain magnetic resonance imaging (MRI) indicated for all subjects with dermatological suspicion of SWS, even in the absence of any neurological signs/symptoms?

It is indicated to perform a brain MRI with gadolinium in case of dermatological suspicion of SWS, preferably after the age of 12 months. Moreover, brain MRI with gadolinium is indicated in all subjects with onset of neurological signs/symptoms with a negative brain MRI performed before the age of 12 months or who never had a neuroimaging. If the patient is asymptomatic and has performed a negative MRI before the age of 12 months, it is preferable to wait the age of cooperation (usually after 6 years) to repeat neuroimaging, thus avoiding sedation.

Whenever possible, in case of high suspicion, a first feed-and-wrap approach (sleep or oral midazolam under monitoring of vital parameters) within 4 weeks of life can be suggested, preferably with a 3 Tesla scanner, avoiding gadolinium injection, for the detection of intracranial involvement, but taking into account the possibility of false negatives.

**Fig. 2:**
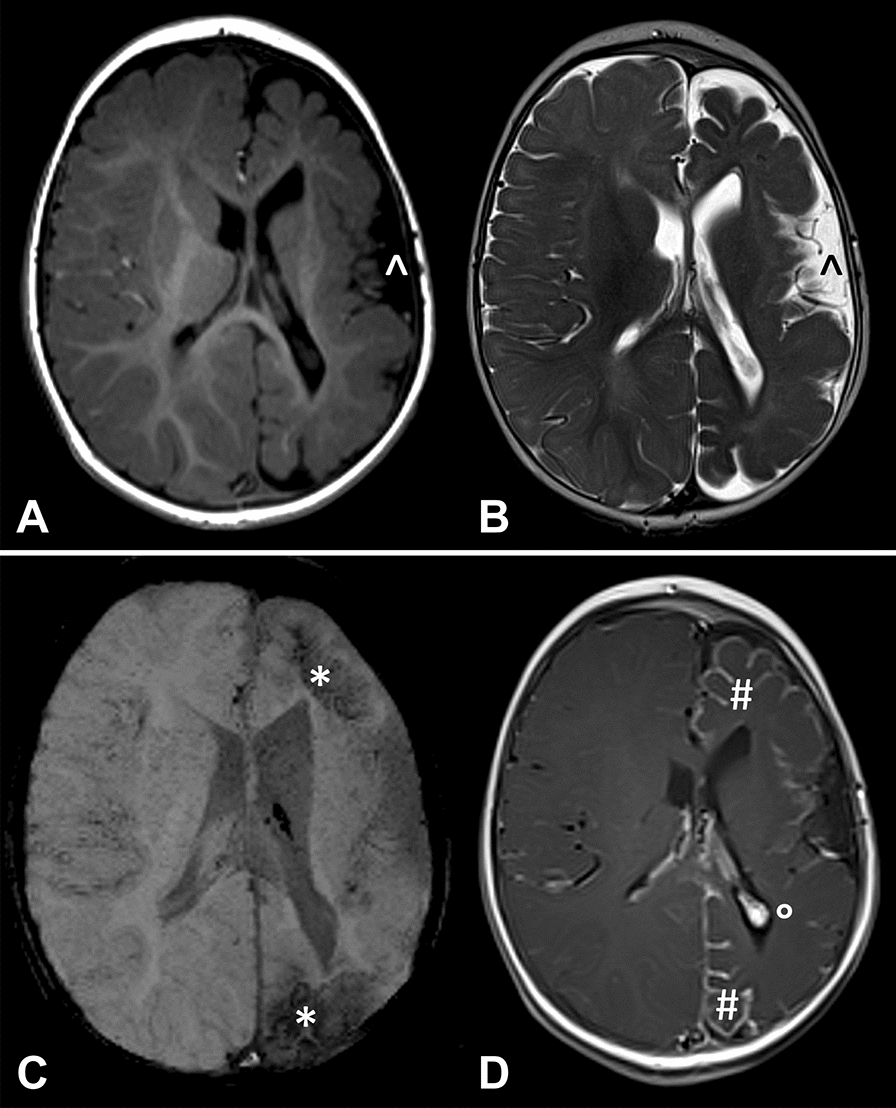
Magnetic resonance imaging (MRI) of the brain in a child with Sturge–Weber syndrome. Axial T1 (**A**), T2 (**B**), susceptibility- (**C**), and gadolinium-enhanced T1 (**D**)-weighted images. Note fronto-parietal atrophy of the left hemisphere with enlargement of adjacent subarachnoid spaces (^); the sharp hypointensity on susceptibility-weighted imaging sequence corresponds to meningeal calcification (*). Pial enhancement over the left frontal and parietal lobes (#), as well as hypertrophied choroid glomus (°) are visible

**Fig. 3:**
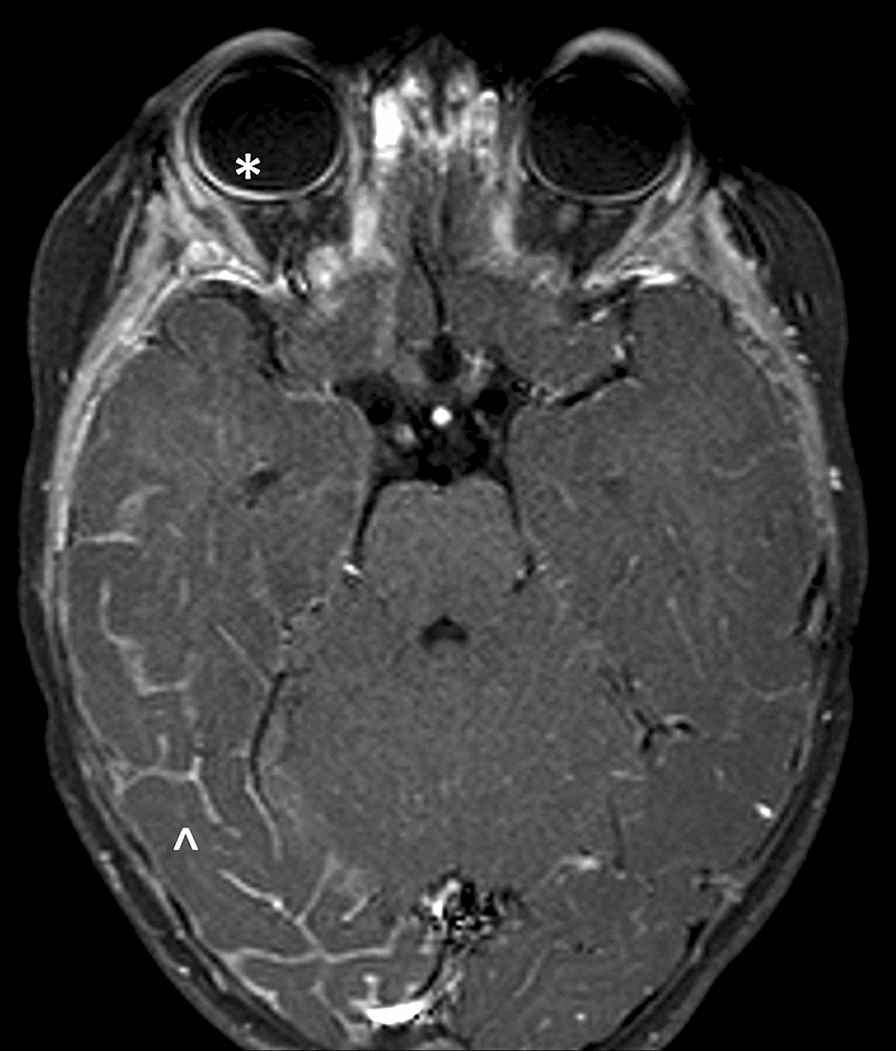
Gadolinium-enhanced axial T1-weighted brain MRI image in a child with Sturge–Weber syndrome. A prominent leptomeningeal enhancement of the right temporo-occipital lobes is evident (^); a homolateral choroidal vascular malformation is also visible into the ocular globe (*), compared to contralateral eye

Contrast-enhanced MRI represents the diagnostic “gold standard” for subjects with SWS, since it shows the presence, extension, and severity of intracranial involvement. Leptomeningeal contrast enhancement may not be visible in the first months of life [54]. However, an ipsilateral choroid plexus enlargement, signal inversion of the white matter and cerebral atrophy represent indirect MRI evidence of cerebral involvement (Fig. 2) [54]. Since it is possible to observe false negatives in early imaging—in particular if brain MRI is performed before 12 months of age—it is preferred to carry out MRI with gadolinium after the first year. Moreover, it is preferable to use a 3 Tesla scanner with dedicated protocols and sequences [among others: diffusion weighted imaging, T2* or susceptibility weighted imaging, venous MR angiography, and high spatial resolution sequences on the orbital region (Fig. 3); after gadolinium injection, it is suggested to perform 3D T1 TSE or 2D T1 SE sequences on the three spatial planes in association to 3D or 2D FLAIR]. It is important to optimize technical parameters of the sequences (TE, TR, and thickness), adapted to infantile ages in which myelination is still progressing. Confirmation of the presence of intracranial involvement even before the onset of neurological signs/symptoms can give the possibility to educate parents for early seizure recognition, avoidance of triggering factors and the use of emergency treatment plans, moreover timing and type of follow-up can thus be tailored to the specific condition.

In third level Paediatric Centres it is now possible to perform brain MRI scans avoiding anaesthesia with the so-called feed-and-wrap approach taking advantage of sleep after feeding or with low-dose oral midazolam [55]. In this case, gadolinium is usually not administered to avoid risk of event awakenings and movements as well as overload on the immature excretory system [56]. Even without gadolinium, it is possible to observe indirect signs of brain involvement such as lateral ventricle choroid plexus hypertrophy (> 5.6 mm), choroid plexus asymmetry, early atrophy, and white/grey matter abnormalities [54, 57], which are present in a high percentage of newborns with SWS [54, 58].

A negative MRI with gadolinium injection performed after one year of age, associated with normal neurological exam and no history of seizure, generally exclude SWS brain involvement [6].

In case of neurosurgical planning, advanced sequences such a diffusion weighted imaging with multiple directions (> 36) or functional-MRI resting state/task can be obtained for the identification of white matter bundles and eloquent areas with better planning of surgery and neuronavigation.

As alternative, advanced MRI sequences can be used for scientific purposes, adapting the protocol targeted on the specific research questions.

#### Is brain computerized tomography (CT) indicated in patients with dermatological suspicion of SWS?

Brain CT is not indicated since it has poor sensitivity, moreover it exposes the patient to a risk associated with ionizing radiation.

CT is less reliable than MRI to identify brain involvement, especially in younger patients in whom calcification and atrophy may still be blurred [7]. CT can be considered in selected cases, depending on the availability of MRI or in emergency in case of other comorbidities (head trauma, hemorrhage).

#### Is neuroradiological follow-up indicated in patients with confirmed SWS?

Control brain MRIs in patients with confirmed SWS are indicated only in case of unexpected worsening of neurological signs/symptoms, with the aim to evaluate the progression of brain involvement (calcifications, atrophy). Control MRIs during acute events such as status epilepticus, migraine or stroke-like episodes can show elements useful to understand the pathogenesis of the event, but do not change the clinical management.

In case of prolonged seizures, cluster of seizures, migraine, epileptic status or stroke-like events, the brain MRI can show transient diffusion and perfusion abnormalities that can help in understanding the pathophysiology of the event but it does not change clinical management, thus, it is not mandatory. Head trauma can be followed by stroke-like episodes; however, neuroimaging is usually unnecessary [59].

#### Is presymptomatic treatment with antiepileptic drugs indicated in children with SWS?

Early presymptomatic treatment with antiepileptic medications may be offered to patients with extensive unilateral or bilateral brain involvement, at higher risk for early-onset epilepsy, after having explained to families their empirical use and possible adverse events.

It is recommended to inform and educate families to the use of rescue therapies in case of prolonged seizures and/or cluster of seizures, associated to appropriate treatment of fever and dehydration.

Early age of seizure onset, frequent seizures and abnormal epileptiform discharges on EEG are associated to poor cognitive and motor outcome in SWS [47, 60]. Moreover, seizures can trigger stroke-like episodes, probably further worsening hypoperfusion of the affected cortical areas. A few small-sized studies with many limitations on presymptomatic use of antiepileptic drugs described better outcomes (cognitive, lesser severity of the epilepsy or in neuroscore index) in the group who received presymptomatic treatment [61, 62]. In this view, the use of presymptomatic anti-seizure medication in children younger than one year could have a positive effect on their development. However, to date there are no controlled randomized studies that can support this approach [63]. In all cases, it is advisable to warn parents and caregivers about the risk of seizure clusters and to encourage the development of individualized emergency plans [7], such as rescue benzodiazepine therapy (e.g. rectal diazepam or oromucosal midazolam) [64, 65].

#### What are the most appropriate anti-seizure medications (ASMs) in patients with SWS and epilepsy?

Antifocal ASMs such as oxcarbazepine, carbamazepine and levetiracetam are considered the first-line therapy. When ineffective, or in patients not eligible for surgery, alternative or adjunctive therapies are lacosamide, lamotrigine, fenobarbital, cannabidiol, topiramate, clobazam, and ketogenic diet. For patients with frequent headaches a shift to valproic acid or lamotrigine should be considered. Home-administered benzodiazepines (buccal/intranasal midazolam and/or rectal diazepam) are first-line agents for acute and prolonged seizures and clustered seizure management. Specialist decisions on pharmacological treatment should be personalized, and consider seizure types, patient age, and possible comorbidities.

Seizure control remains a priority for patients with SWS, and ASMs are the first-line treatment for SWS-related epilepsy [53, 66]. Many individuals with SWS experience focal-onset seizures, and oxcarbazepine/carbamazepine and levetiracetam are a first-line ASMs [66–70]. Oxcarbazepine may be preferable to other anticonvulsants due to fewer side effects [68]. Other ASMs reported effective in patients with SWS and epilepsy are usually administered as lacosamide, phenobarbital, lamotrigine, cannabidiol, clobazam, and topiramate [69–71]. As to side effects, topiramate does not appear to cause new onset or exacerbation of existing glaucoma [68]. Both carbamazepine and oxcarbazepine can be associated with thyroid insufficiency, which may exacerbate hypothalamic-pituitary dysfunction sometimes observed in patients with SWS [63].

Recently, the mTOR inhibitor sirolimus has been reported in case series as possibly effective in reducing seizure frequency in drug-resistant epileptic patients [72].

#### What are the most appropriate drugs in case of headache or migraine?

At present, there are no guidelines on acute and preventative management of headache. In most countries, sleep, hydratation, ibuprofen, paracetamol and antiemetics are used. It is important to consider that in younger patients with cluster of seizures associated to irritability/neurological signs, analgesic treatment combined to a short-term benzodiazepine pulse should give relief and shorten the acute phase. Data from the literature confirm that triptans are safe and frequently effective. The most used drugs for prophylaxis are anti-seizure medications (valproate, lamotrigine, topiramate or gabapentin) and flunarizine.

Headaches are frequent in patients with SWS, mostly in older children and adults, often impairing their QoL [73]. Even if there are no guidelines for acute or preventative management, in most countries, sleep, hydration, ibuprofen, paracetamol and antiemetics are used [67]. Data from the literature confirm that triptans are safe and frequently effective [73]. The use of anti-seizure medications for migraine prophylaxis can improve both seizures and headache/migraine attacks.

#### Is acetylsalicylic acid (ASA) indicated?

Despite the absence of evidences, low-dose ASA (3–5 mg/kg/day to a maximum of 100 mg/day) should be offered early in the disease course, after the appearance of neurological signs/symptoms, in patients with extensive brain involvement and/or cluster of seizures associated to subsequent neurological worsening.

One of the hallmarks of SWS in the central nervous system is abnormality in the medullary and subependymal veins as well as deep venous structures associated with impaired venous outflow [74]. Venous congestion, stasis, and thrombosis predispose SWS patients to ischemia-related progressive brain injury. Thus, low-dose ASA (3–5 mg/kg/day) has been considered for management of seizures and stroke-like episodes [75, 76].

At present, there are no placebo-controlled randomized trials that determine the real efficacy of ASA as preventative treatment, but retrospective studies and reports of small series show a reduced incidence of stroke-like episodes and seizures in patients treated with ASA [75]. It is unknown if this could be related to a better neurodevelopmental and functional outcome. In a small sample of patients with presymptomatic use of low-dose aspirin (prior to the onset of seizures or strokes) the range of outcomes by neuroscore a few years later was variable [66], so definite conclusions if early treatment can modify disease course cannot be drawn.

The side effects of low-dosage aspirin are very scarce and usually mild, such as increased bruising and nosebleeds [75], so the potential benefit probably outweighs the risks [6]. The use of ASA in SWS is likely more extensive in the United States compared to Italian experience (US: 49% patients treated with ASA) [69].

#### When is a specialist evaluation for the surgical treatment of epilepsy indicated?

Surgical indication should be reasonably considered in drug-resistant patients, mostly in subjects with early onset of seizures and pre-existing developmental impairment and/or neurological deficit. We cannot suggest any specific type of surgical approach since every centre for epilepsy surgery has its proper techniques.

Type of surgery (hemispherotomy, sublobar/lobar resections, multilobar disconnections) depends on the extension of the epileptogenic area and its relationship with functional areas, and has to be always evaluated in a third level centre for epilepsy surgery.

Early surgery should have a favourable role on cognitive and motor outcome. In selected cases with bilateral involvement, hemispherotomy on the more active hemisphere should be considered, with palliative intent.

Drug-resistant epilepsy in SWS probably affects 20–25% of patients [9, 77]. Surgical approach, in particular the type and extension of the surgery, depends on the extension of the epileptogenic area and its relation to functional eloquent areas.

A recent meta-analysis shows that hemispherectomy and hemispherotomy are invariably followed by hemiparesis [78]. Thus, hemispheric surgery is suggested preferably during the first 4 years of age, or in patients who already present significant deficits for functions that can be affected by planned surgery [79–81]. According to other authors, surgery should be considered earlier in cases in which progressive hemiparesis and/or neurocognitive deterioration are expected [82–88]. In most limited brain involvement, it is possibile to proceed with sublobar/lobar resections [89, 90] or combined tecniques with resections and disconnections. Wide multilobar disconnections are indicated in intermediate cases [80, 81, 91, 92].

In patients with bilateral leptomeningeal vascular anomalies and severe drug-resistant epilepsy affecting the QoL, surgery (hemispherotomy on the more epileptic side or vagus nerve stimulation) can be considered and proposed with palliative intent only in well-selected cases [82]. A recent meta-analysis failed to identify a statistically significant difference in the epileptological outcome comparing resection and hemispherotomy (Engel class I, i.e. patients free from seizures, 69.2% vs. 87.3%) [78].

#### Do children with SWS need psychomotor/psychodiagnostic screening?

Considering the high prevalence of neurocognitive and behavioural comorbidities in children with SWS, a standardized screening of their evolutionary trajectory can lead to early rehabilitative treatment. A developmental/neurocognitive evaluation is recommended at diagnosis, at seizure onset, in case of suspected neurodevelopmental delay and appearance of neurocognitive/behavioural abnormalities.

The primary care paediatrician should regularly supervise children’s development during follow-up. In case of suspected developmental delay, it is recommended to refer the patient for neurodevelopmental or neurocognitive evaluation using standardized scales (e.g. Bayley, Griffiths) with the aim to start rehabilitation therapies and an individualized educative plan.

Given the high prevalence of neurocognitive/behavioural comorbidities in children with SWS, the screening of their developmental and neuropsychiatric status is critical in order to initiate early interventions, especially since parents may not recognize subtle developmental delays [63]. Intellectual disability affects almost 60% of patients [7]. Attention deficit and hyperactivity disorder, learning disorders, as well as other neuropsychological or behavioural issues are frequent [93]. Recent studies suggest also that SWS patients may be at higher risk of autism spectrum disorder [94]. Proper intervention can improve clinical outcomes.

#### What is the psychological impact of SWS on patients and their parents and how it should be managed?

Psychological implications of SWS are widely underexplored and underestimated both in early ages (neurodevelopmental disorders, emotional-behavioural issues, and learning difficulties) and in adulthood (mood disorders, low self-esteem, emotional disorders, social isolation).

In case of clinical doubt of discomfort or psychological issues, it is recommended to refer the patient for a psychodiagnostic evaluation. This assessment can establish the need of psychotherapy or welfare/social support, offer parents’ counselling, and evaluate the aesthetic impact and the stigma perceived by patients and/or their families.

Psychological dysfunction may occur in 50% of adult patients, including depression, anxiety, low self-esteem, shame, emotional dysregulation, and isolation [7]. Although the disease psychological impact is underexplored, it appears to be one of the most unmet needs for patients and parents. Suicidal risk is concrete and must be evaluated carefully also in drug choices [95].

### Ophthalmological recommendations

#### In infants with a CM suggestive of SWS, what parameters other than intraocular pressure (IOP) are relevant for the diagnosis of glaucoma?

The ocular parameters relevant for the diagnosis of glaucoma are, in addition to the IOP, the variation of the corneal diameter, axial length and cup-to-disc ratio. Blepharospasm, tearing and photophobia are also suspicious symptoms.

**Fig. 4:**
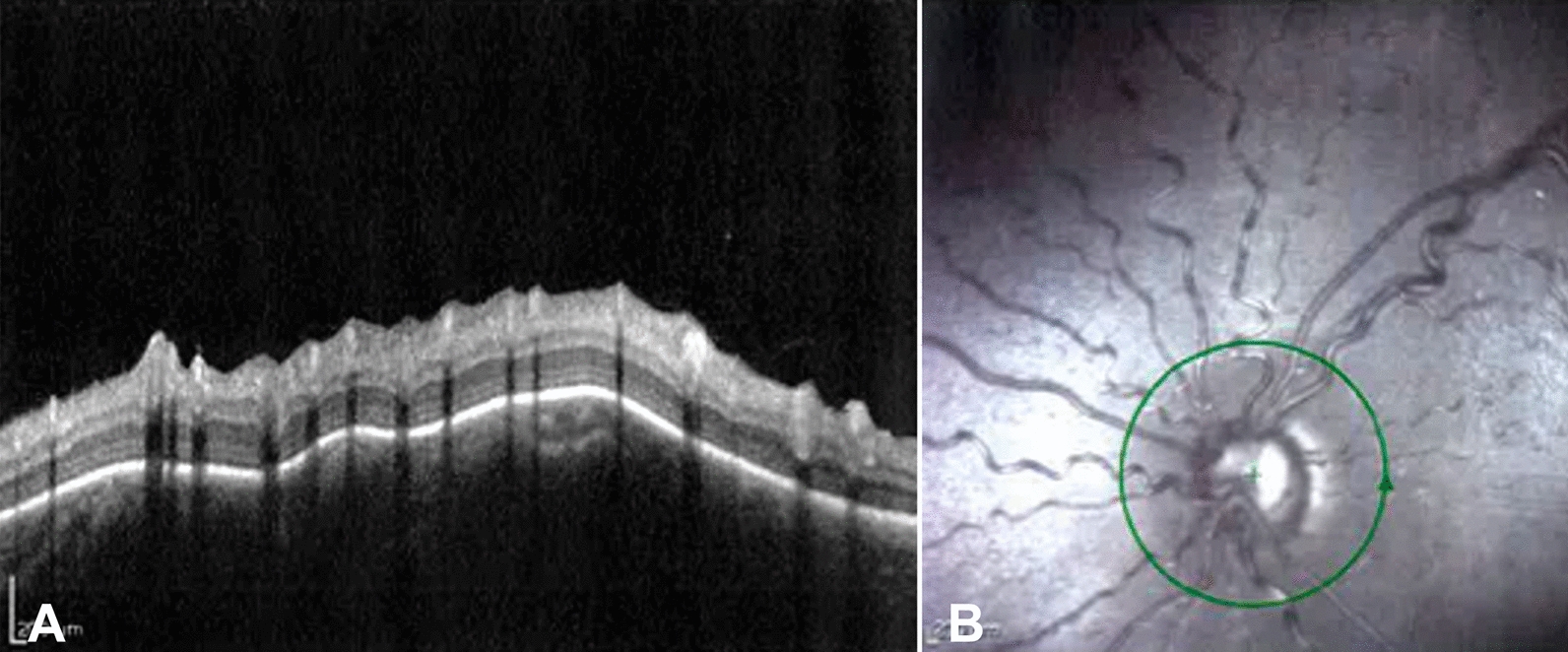
Optical coherence tomography of a patient with Sturge–Weber syndrome presenting ocular vascular malformation (**A**) showing a large glaucomatous excavation of the optic nerve and vascular congestion from increased episcleral venous pressure (**B**)

Approximately 50% of SWS patients have an ocular abnormality, usually ipsilateral to the vascular malformation [13, 96]. Vascular abnormalities may involve the eyelid, conjunctiva, episclera, and/or retina and choroid (Fig. 4). In addition, there is a lifelong risk of developing glaucoma, which is one of the preventable causes of vision loss. Glaucoma associated with SWS can have early (60% in childhood) or late (40% in adulthood) onset [97]. Early onset is typically associated with angular abnormalities, like congenital glaucoma due to anterior segment dysgenesis, late onset is secondary open-angle glaucoma, usually due to increased episcleral venous pressure [13, 98, 99].

If vascular malformations compatible with SWS are detected, the newborn should undergo a complete ophthalmological examination, to primarily evaluate the extension of the malformation, the presence of eyelid involvement and the subsequent possible ptosis. The examination of the anterior segment will allow to assess the transparency of the cornea and lens. In case of glaucoma, corneal enlargement and opacity may be present. Pupil dilation will allow to evaluate the optic nerve, the vessels and the possible presence of retinal alterations and choroidal vascular malformations [100, 101]. In the absence of pathological or suspicious ocular signs, complete ophthalmological examination can be repeated annually [102]. In newborns in whom glaucoma is suspected, evaluation under inhalation anesthesia should be performed for easier exploration, IOP assessment, transverse corneal diameter and axial length measurement [99]. These indices are used to monitor ocular growth, which can be abnormal in case of glaucoma secondary to SWS. The Goldmann applanation tonometer is considered the gold standard for measuring IOP in children [98]. The previous tests should be performed two or three times a year until the disease is controlled and it is possible to carry out the monitoring tests on an outpatient basis [7]. In case of ptosis, re-evaluation at 3–6 months for possible development of amblyopia is indicated [103].

#### Can fundus examination reveal retinal changes? When can the use of diagnostic retinal imaging be justified and with which tools?

Fundus examination shows retinal alterations related to choroidal vascular malformations in many cases. Diagnostic imaging should be performed to identify and monitor specific ocular anomalies. Optical Coherence Tomography (OCT) is used to define the extent of the lesion and its location, and angiography in suspected exudation. Ultrasound is used to define the reflectivity of the lesion; in children or in non-cooperative patients, it can be performed under anesthesia. Fundus photography with possible angiography is employed in children or non-cooperative patients, in the suspicion of exudation.

The retinal changes associated with SWS are related to diffuse or localized choroidal vascular malformations. Choroidal malformations are typically unilateral, but can be bilateral in up to 10–30% of patients [101, 104]. They can be observed on the fundus as a localized orange lesion (difficult to recognize in non-compliant patients) or as a markedly red fundus (“tomato ketchup” appearance), in case of a diffuse lesion [104–106]. If localized, they can be usually identified by clinical examination of the fundus oculi [105]. In newborns, examination under deep sedation can be useful. OCT is the preferred and gold standard imaging technique for analyzing the structure and reflectivity of the retina and choroid. It is a non-invasive and relatively quick technique that can help define the characteristics of retinal and choroidal lesions. In addition, indocyanine green angiography can reveal the presence of choroidal lesions and vascular leakage. Ultrasound scans can show high internal reflectivity and a solid lesion with increased echogenicity within the thicker choroid (diffuse choroidal thickening) [107]. MRI with gadolinium is useful for evaluating a sickle-shaped choroidal lesion, which appears thicker at the posterior pole and thinner near the ciliary bodies [108].

#### Is it advisable to maintain ophthalmological follow-up in patients with SWS life-long?

Since the risk of developing glaucoma is present throughout life, monitoring must be regular and continuous. It is advisable to carry out at least annual outpatient check-ups from birth, in the absence of complications. The follow-up will then be personalized, for example in case of amblyopia, glaucoma or retinal vascular malformation. Tonometry will be performed under anesthesia until the patient is cooperative and can undergo outpatient check-up.

**Fig. 5:**
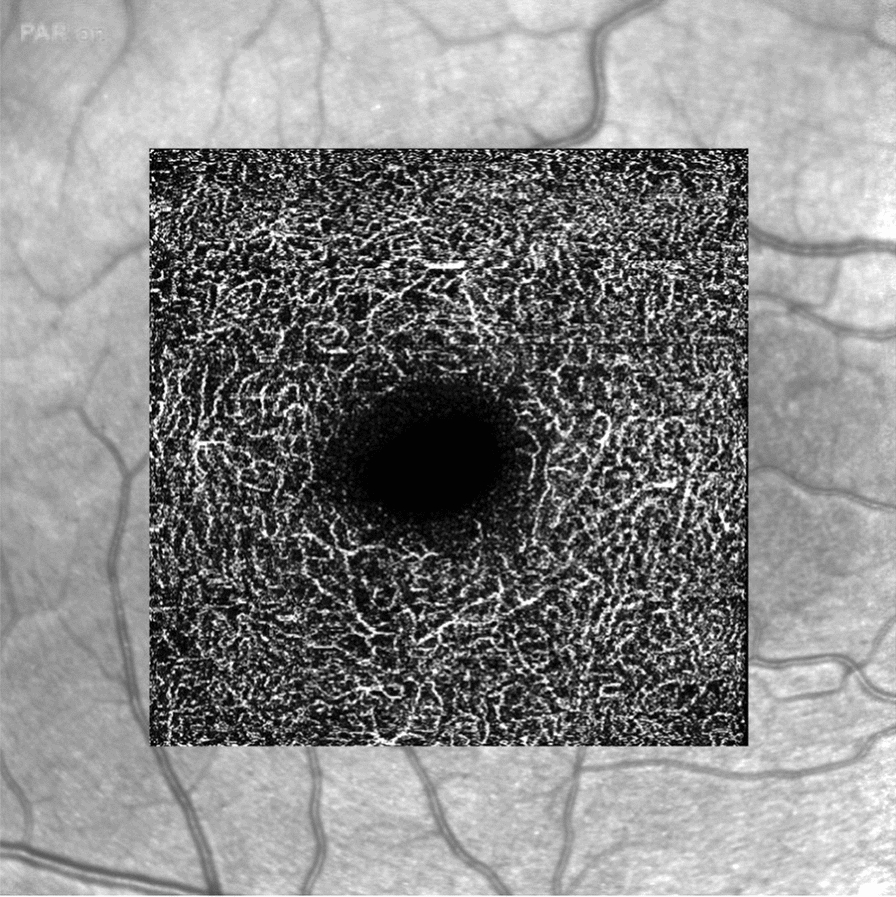
Angio-ocular coherence tomography image of a Sturge–Weber patient showing congestion of the deep vascular plexus

As the risk of glaucoma development is present throughout life, monitoring must be continuous [13]. In pediatric patients, the main risks include the development of amblyopia and glaucoma. In uncomplicated children, annual eye monitoring is sufficient [109]. However, in the presence of amblyopia or glaucoma, personalized follow-up schedule should be implemented based on the individual's clinical risk [103]. Tonometry will be performed under inhalation anesthesia until the patient is cooperative, then in an outpatient setting [110]. A complete pediatric ophthalmologic assessment should also include evaluations of visual acuity and cycloplegic refraction [111]. In adulthood, the main complication is glaucoma development, and annual or more frequent follow-ups should be scheduled based on the clinical risk [112]. Visual field testing is crucial in adults, as it allows for the assessment of the extent and progression of perimetric damage, which refers to visual loss due to ocular hypertension-dependent optic nerve damage. In addition, anatomical damage of the optic nerve in adults can be monitored using OCT (Fig. 5).

#### Does pharmacological therapy play a role in the management of glaucoma in patients with SWS?

Infantile glaucoma usually requires surgical therapy; medical therapy is used only in the initial phase of the disease, pending intervention. In adult glaucoma, pharmacological therapy can be used, depending on the degree of ocular hypertension. The international guidelines for the management of glaucoma should be followed.

The objective of medical therapy for glaucoma is to decrease IOP, which is the primary cause of optic disc damage. In the case of adult glaucoma, initial treatment typically involves the administration of one medication or a medication combination. Glaucoma therapy entails the use of topical drugs (eye drops) from various classes, including beta-blockers, carbonic anhydrase inhibitors, prostaglandin analogues, and alpha-2-agonists [112]. If the desired IOP levels are not achieved with the appropriate medical therapy, and there is evidence of worsening perimetric damage or an unfavorable impact on the patient's QoL, surgical intervention should be considered [112–114].

#### What are the indications for surgery and which type of surgery is most suitable for the treatment of glaucoma in SWS?

Surgery is indicated when IOP is not controlled despite appropriate and maximal pharmacological therapy, due to intolerance or allergy to hypotonic medications, or therapy non-compliance. Surgery involves penetrating filtering procedures for glaucoma. Retro equatorial drainage systems are used as a last choice.

The goal of glaucoma surgery is to create an alternative pathway for the outflow of aqueous humor. Penetrating filtering procedures are goniotomy, trabeculotomy, trabeculectomy, combined trabeculotomy–trabeculectomy, deep sclerotomy and glaucoma drainage devices that have all shown effectiveness in controlling IOP in SWS patients [114–116]. Parasurgical laser therapy (argon laser and selective laser trabeculoplasty) is not applicable [112]. Retro equatorial drainage systems are used as last choice [115, 117, 118].

#### When is laser treatment indicated for the treatment of retinal vascular malformations?

Laser treatment of choroidal vascular malformations should be performed when there is a risk of complications such as subretinal hemorrhage, serous retinal detachment, cystoid macular edema and neuroepithelial detachment.

The goal of treatment for choroidal vascular malformations is to induce involution of the lesion, with reduction of subretinal and intraretinal fluid and minimal disruption of the sensorineural retina [119]. The decision to treat choroidal vascular malformations should be based on the potential for visual acuity and the extent of detachment. The management of choroidal lesions can be very challenging and treatment options may be limited because both localized and diffuse lesions often involve juxtapapillary and subfoveal sites. Argon laser photocoagulation usually leads to poor visual acuity, a high rate of recurrent subretinal exudates requiring more photocoagulation, and an elevated percentage of retinal detachment [120, 121]. It can also lead to irreversible scotoma when treating lesions near the optic disc or macula.

### Dentistry recommendations

#### When should the patient with SWS be referred to the dentist and how should follow-up be planned?

The first dental assessment should be performed as soon as the diagnosis of SWS has been made, in order to promptly evaluate possible mucosal vascular lesions, dental alterations in terms of size and/or timing of tooth replacement, and skeletal asymmetries.

If there are no lesions in the oral mucosa, follow-up should be every six months.

If lesions are present, follow-up should be every four months to assess periodontal health status, tendency to spontaneous bleeding, degree of gingival hypertrophy, and any skeletal and functional consequences.

Oral manifestations have been reported in 40–50% of SWS patients [122, 123]. They may be minimal or wide and complex and consist in modest or severe soft tissue hypertrophy and/or nodules within the CM [122, 123]. The uncontrolled growth of these nodules can cover the dental crowns, even completely, causing impaired chewing [124, 125]. Moreover, some anti-seizure medications may cause gingival hyperplasia [122, 123], which determines greater difficulty in cleaning the dental elements. In addition, plaque accumulation contributes to increased inflammation with further gingival hypertrophy and formation of pseudopockets [126].

**Fig. 6:**
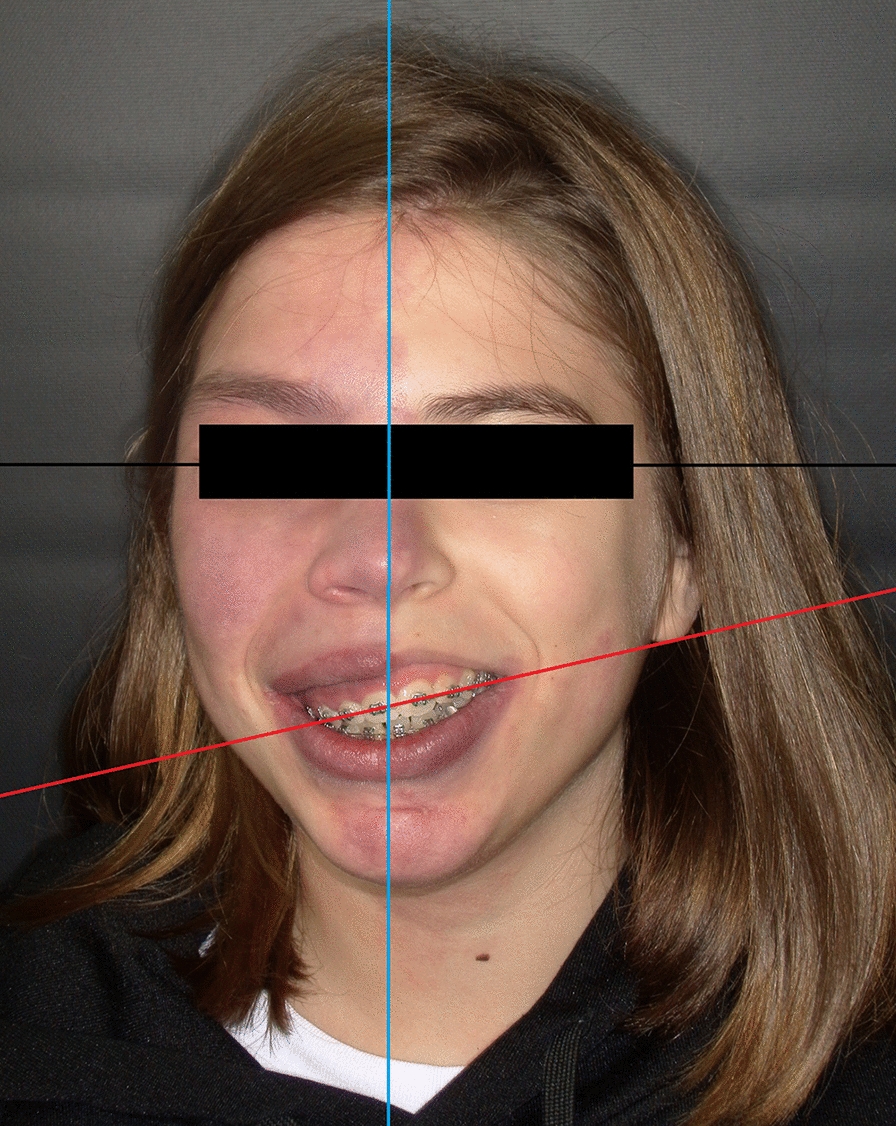
Clinical features of an adolescent affected with Sturge–Weber syndrome presenting a capillary malformation with hemifacial hypertrophy. Note the relationship between the bipupillary plane (black line) that runs parallel to the background and the inclination of the occlusal plane (red line), secondary to upper jaw asymmetry

These patients can present spontaneous hemorrhages, sustained by neoangiogenesis [125], and/or induced by masticatory trauma [122]. Moreover, altered timing of tooth replacement, macrodontia and a different skeletal growth pattern on the side of the CM are reported. The subsequent asymmetry causes different malocclusion patterns [127] (Fig. 6).

A model follow-up for SWS patients is shown in Table 1. However, the planning should be individualized considering patient cooperation level and disease manifestations [128, 129].

**Table 1:** Oro-dental follow-up timing and evaluations in patients affected with Sturge–Weber syndrome

Age	Oral involvement	Follow-up timing	Focal points
0 to 6-year-old	No	6 months	Deciduous dentition, oral hygiene, presence of caries
	Yes	3–4 months	Mucosal lesions, deciduous dentition, oral hygiene, presence of caries
6 to 12-year-old	No	6 months	Dental replacement, oral hygiene, presence of caries and malocclusion
	Yes	3–4 months	Mucosal lesions, dental replacement, oral hygiene, presence of caries and malocclusion
> 12-year-old	No	6 months	Permanent dentition, periodontal probing, presence of caries and malocclusion
	Yes	3–4 months	Mucosal lesions, permanent dentition, periodontal probing, presence of caries and malocclusion

#### What are the age-related dental assessments of SWS patients?

0–3-year-old: investigate the presence of any vascular lesion in the oral and perioral soft tissues, assess facial symmetry and the status of eruption of the deciduous series. Establish a correct oral prevention and prophylaxis protocol by age, according to national guidelines.

3–6-year-old: carry out the evaluations listed above and monitor the first phase of tooth replacement and any jawbone asymmetry.

6–12-year-old: carry out the assessments listed above and monitor the transition from mixed to permanent dentition to early detect any abnormalities. Perform an initial panoramic radiography. Assess periodontal health status. Investigate any malocclusion related to a skeletal asymmetry ipsilateral to the CM and lingual posture.

> 12-year-old: monitor vascular lesions and perform orthodontic evaluation of dental occlusion. If needed, joint assessment with the maxillofacial surgeon to discuss indications to pre-surgical orthodontics or skeletal compensation. Assess periodontal health status.

Dental assessment must consider specific aspects depending on patient's age. Early diagnosis of oral manifestations allows to reduce the number of investigative procedures that often have to be performed urgently. The evaluation parameters concern the extent of the CM, the presence of soft tissue hypertrophy, the dental element number in the arch, and the morphology of the oral cavity. It is important to consider the synergic effect of (i) the plaque-induced hypertrophy, (ii) the hyperplasia secondary to antiepileptic medications, and (iii) the development of hypertrophy of the oral mucosa affected by CM. The performance of repeated non-surgical periodontal therapies and education to regular oral hygiene are fundamental [129, 130]. Various conservative and extractive interventions of the elements involved in the CM are described [131, 132].

**Fig. 7:**
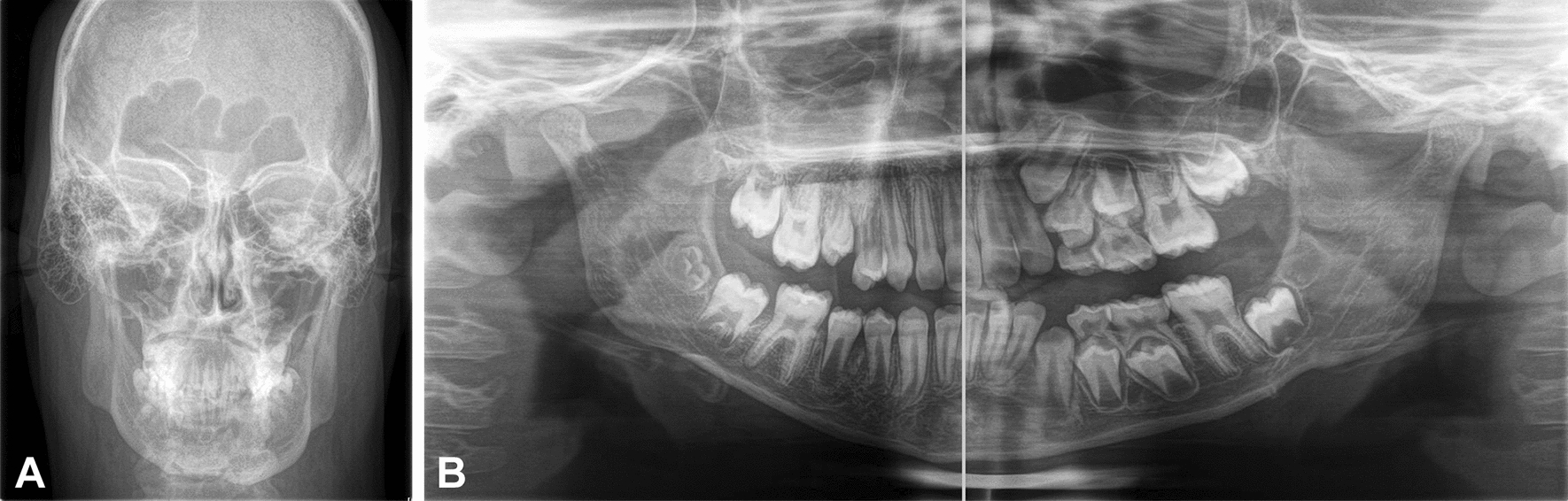
X-ray images of two patients affected with Sturge–Weber syndrome. In **A** the skull antero-posterior view of an adolescent shows asymmetry of the frontal sinus, petromastoid region of the temporal bone, and mandibular inferior margin. **B** Dental panoramic radiography of an 8-year-old child with a right hemifacial capillary malformation. Note the asymmetry in deciduous tooth replacement between the right and left side

The need for selective embolization of the maxillary arteries prior to an extraction is reported [133]. Perilesional plexus anaesthesia is performed with a needle changed at each infiltration [134]. In case of surgical incision, suture is always recommended to promote better healing [121]. In more extensive surgical procedures, blood transfusion may become necessary [121]. Asymmetrical growth of the soft and skeletal tissues with dentobasal discrepancies must be carefully evaluated to propose an appropriate therapeutic plan [126, 135, 136]. A screening radiograph in the early phase of tooth replacement is recommended in order to detect abnormalities not visible on clinical examination (Fig. 7).

Orthodontic therapy must be planned according to the patient’s growth phase and compliance. It is not always possible or desirable to achieve dental compensation. A multidisciplinary approach with the maxillofacial surgeon must be performed.

#### Can laser treatment be effective in the management of vascular lesions of the oral cavity in SWS patients?

Laser treatment appears to be an effective therapy for the management of vascular lesions of the oral mucosa. It allows gingivectomy of the hypertrophic component and non-surgical periodontal therapy through decontamination of the gingival sulcus. The choice of the type of laser depends on the treatment to be performed.

With the advent of laser technology, surgical treatments have become easier due to laser advantages: no need for sutures, less surgical invasiveness, reduced bleeding tendency, and improved post-operative pain control, with an overall better compliance and a reduced cost/benefit ratio. Thus, at present, laser treatment represents the gold standard. The type of laser varies from the CO2 laser (10,600 nm) to the neodymium-YAG (Nd:YAG) laser (1,064 nm) and the diode laser (808–980 nm). Gingivectomy can be performed with Nd:YAG laser at frequency of 40 Hz, energy of 130 mJ, and power of 4 W. It has been reported that Nd:YAG lasers with powers from 4 to 6 W cut efficiently small areas of mucosa with a greater coagulative action than the CO2 laser; the latter, however, showed a sharper and faster cut [123, 127, 137].

Diode laser, using a 300 μm disposable tip in a 1.5–3 W continuous mode of contact, allows to remove gum tissue and to expose adequate amounts of tooth structure. In addition, gum groove bacterial decontamination can be achieved through topical antiseptics activated by the diode laser fiber applied at low power (1 W) inside the gum pocket [121, 138, 139].

### Plastic surgery recommendations

#### When is orthognathic surgery indicated in patients with SWS?

Based on recent literature, patients affected by SWS who underwent orthognathic surgery show satisfactory results, comparable to standard patients. Subjects with SWS who have adequate neuro-cognitive development and therefore can ask for treatment, can undergo orthognathic surgical treatments for the correction of dento-skeletal malocclusions.

Among its clinical manifestations, SWS presents soft tissue hypertrophy and skeletal disorders of the maxilla, therefore patients may present a higher incidence of malocclusions [19, 140]. In the past, no surgery was performed in these patients due to intraoperative risk of bleeding. In the last years, several cases with skeletal basis correction through orthognathic surgery have been reported with satisfying results [130, 141–143]. Patients with SWS can undergo orthognatic surgical treatments following the indications for the healthy patients [144].

#### What is the role of plastic surgery in patients with SWS and CM associated with tissue hypertrophy?

Surgery in patients with SWS is nowadays believed to be safe and effective with only a modest increase in bleeding risk. Indications for surgery include debulking of CM areas with significant tissue hypertrophy (especially the lip), removal of major or minor nodules (when the scarring will be more acceptable for the patient than the lesion), or recurrent bleeding.

The aim of plastic surgery in patients with SWS is to minimize the social impact of malformation, and to reduce growing nodules and soft tissue hypertrophy [145, 146]. The gold standard for the prevention and treatment of proliferating nodules is laser therapy, but nodules may be unresponsive and the effectiveness of laser treatment for tissue hypertrophy remains debated [12, 146]. Nevertheless, treatment plan choice should take into account that tissue overgrowth is related to activating somatic mutations. Surgical treatment, which was deemed contraindicated for bleeding risk, is nowadays considered safe and reliable [143, 146, 147].

Zonal approaches—based on aesthetic subunits and skin tension lines—have been proposed for debulking of major nodules within CMs [141, 148, 149]. Depending on the number of areas involved, multiple step approaches may be performed. It will be obviously essential to provide psychological support for the patient to face such a complex surgery.

### Primary care paediatrician recommendation

#### Which is the role of the primary care paediatrician (PCP) in the care of children with SWS?

If the PCP suspects SWS, he/she should refer the child to a Reference Centre. For children with confirmed SWS, the PCP should follow the patient and family in accordance with the Reference Centre. The role of the PCP is fundamental to ensure the best QoL and appropriate patient management by caregivers.

The PCP should monitor child growth and neurodevelopment, identify signs/symptoms of suspect hypothyroidism, and/or growth hormone deficiency.

In addition to cutaneous, neurological, ophthalmological, and oral manifestations, an 18-fold increased relative risk of growth hormone deficit in paediatric patients with SWS has been reported [150]. It usually occurs in absence of specific neuroradiological abnormalities. Moreover, central hypothyroidism is more frequent in children with SWS [151]. Since both conditions deserve medical treatment, it is important to monitor the patient during follow-up visits, referring him/her to an endocrinologist in case of signs/symptoms and/or abnormalities in the auxological parameters. No contraindications to vaccine administration have been reported; some authors suggest to perform annual flu vaccine [152].

Depending on the National Health System rules, paediatric patients will be followed by the PCP or by the general practitioner who will be in charge of the global patient management, including referral to the SWS reference centre specialists and subsequent follow-up contacts.

## Discussion

SWS is a complex disorder that requires the multidisciplinary involvement of healthcare professionals specifically trained and skilled to guarantee the better outcomes. SWS care still presents unsolved issues concerning the most appropriate management and treatment strategies and modalities. Indeed, there is no definite evidence in the literature about the best timing to perform brain MRI, which is a necessary radiological exam to evaluate cerebral involvement [6, 54]. Moreover, there are only limited retrospective studies on presymptomatic treatment with anti-seizure medications in case of extensive brain involvement [61–63]. Finally, there are different opinions about performing PDL treatment of CMs under general anaesthesia/sedation, in particular during the first year of life [38–43]. These are only some examples of key questions that would require longitudinal studies to be clarified.

This manuscript is the result of the first Italian multicentre consensus on the management of SWS, involving experts from three Reference Centres, who are also members of the scientific committee of the Italian Sturge–Weber Association.

Since SWS is a systemic disorder, the involvement of a multidisciplinary group has been crucial to consider all disease aspects, including those related to oral cavity assessment, care and follow-up, and facial tissue hypertrophy and skeletal involvement treatment. The patients may be followed in different settings depending on their needs and hospital organization. In a similar way, various modalities for patient visits by the members of the multidisciplinary team can be adopted, provided that each case is jointly discussed and the treatment and follow-up plan agreed upon by the group. This consensus, based on a Delphi methodology, allowed to define practical recommendations for SWS global care, useful to uniform clinical practice in reference centres, in other hospitals, and in outpatient settings where SWS patients are followed. Though developed by Italian reference centres, this consensus could be translated also to other countries, such as European ones. Indeed, following the Council Recommendation of 2009 [153], European Member States have established and implemented specific plans for improved care for patients affected with rare diseases.

Specifically, consensus has been reached regarding the following controversial issues: (i) timing of MRI in asymptomatic patients, which has been recommended after the age of 12 months, (ii) presymptomatic use of anti-seizure medications which may be offered only in case of extensive disease at high risk for early-onset epilepsy, (iii) PDL treatment for CM to be performed preferably under sedation in the first year of life. However, some statements are based on expert opinion due to insufficient literature evidence. An additional limitation of the present consensus is that not all Italian Reference Centres have been involved in the study.

In conclusion, additional clinical research and prospective trials are necessary to generate solid evidence as the basis for clinical practice guidelines.

## Supplementary Information

Additional file 1.

## Data Availability

Not applicable as no datasets were generated or analysed during the present study.

## Abbreviations

ASA: Acetylsalicylic acid
ASM: Anti-seizure medication
CM: Capillary malformation
CT: Computerized tomography
EEG: Electroencephalogram
ERN: European reference network
IOP: Intraocular pressure
MRI: Magnetic resonance imaging
NdYag: Neodymium-yag
OCT: Optical coherence tomography
PDL: Pulsed dye laser
QoL: Quality of life
SWS: Sturge–Weber syndrome
PCP: Primary care paediatrician
