# Supplementary material for: Multidisciplinary, multicenter consensus for the care of patients affected with Sturge–Weber syndrome
Source: Orphanet J Rare Dis. 2025 Jan 16;20:28. doi: 10.1186/s13023-024-03527-w (PMC11740666; doi:10.1186/s13023-024-03527-w)
Supplement: Supplementary file 1 — Additional file 1. [file 13023_2024_3527_MOESM1_ESM.docx]

**Table S1**. **List of recommendations by specialty and level of agreement**

| 1. **Dermatological recommendations** | **Level of agreement** |
| --- | --- |
| 1. **Which distribution of the capillary malformation (CM) on the face should lead to the suspicion of Sturge-Weber syndrome (SWS)?**   *CMs of the face involving the frontal region, bordered inferiorly by a line joining the outer canthus of the eye to the upper portion of the ear and encompassing the upper eyelid, as well as CMs of the middle frontonasal region should suggest suspicion of SWS.* | 91% (30/33) of experts rated this recommendation ≥3 on a Likert scale at first round |
| 1. **What is the most appropriate age to start pulsed dye laser (PDL) treatment?**   *The most appropriate age to start PDL treatment for CM is early childhood. Early treatment improves the effectiveness of therapy, and quality of life (QoL) of the patient and parents.*  *It is currently correct to inform parents of the two approaches, in sedation or with anesthetic cream, and their related risks and benefits. The use of sedation, reducing the frequency of sessions, is recommended to avoid physical and psychological suffering and its consequences. In children, sedation is always necessary for treatment of the orbital region.* | 88% (29/33) of experts rated this recommendation ≥4 on a Likert scale at first round |
| 1. **Is PDL without sedation recommended in the first year of life?**   *Exposing the infant to pain is inadvisable. In contrast, the use of sedation/general anesthesia under the age of one year for PDL treatment of CM is a practicable and preferred strategy. Sedation should be considered as part of a course of care shared by a multidisciplinary team and individualized with respect to the patient's clinical condition and needs.* | 91% (30/33) of experts rated this recommendation ≥4 on a Likert scale at first round |
| 1. **Which is the recommended sedation modality to perform PDL?**   *Sedation of a patient with SWS should be chosen based on a careful medical history and a complete clinical assessment. In the absence of neurological symptoms in the past 3 months, the anesthesiologic approach is not different from that adopted in the healthy pediatric population of comparable age. In patients with neurological symptoms that cannot be controlled by therapy, sedation is recommended with a post-procedural observation of at least 24 hours. The combination of topical anesthetic cream with airway administration of sedative medications provides the best conditions for treatment of uncooperative patients. A recent (within the last 3 months) electroencephalogram (EEG) tracing in patients on anticonvulsant therapy is recommended.* | 91% (30/33) of experts rated this recommendation ≥3 on a Likert scale at first round |
| 1. **Neurological recommendations** |  |
| 1. **Are neurological visit and electroencephalogram (EEG) recommended for children with clinical suspicion of SWS?**   *Any child with facial CM at high-risk for SWS should be referred to a specialist expert in pediatric neurology for a baseline evaluation (clinical history and neurological examination) and EEG (during sleep, better if 30 minutes, at least 8 channels), preferably during the first three months of life.* | 91% (30/33) of experts rated this recommendation ≥4 on a Likert scale at first round |
| 1. **Are follow-up neurological visits and EEGs indicated for patients with confirmed SWS?**   *Longitudinal follow-up with clinical neurological evaluations and EEGs is indicated for infants with confirmed SWS, preferably every 6 months during the first 2 years of life for parental education and monitoring of neurologic red flags and seizures.* | 88% (29/33) of experts rated this recommendation ≥4 on a Likert scale at second round |
| 1. **Is brain magnetic resonance imaging (MRI) indicated for all subjects with dermatological suspicion of SWS, even in the absence of any neurological signs/symptoms?**   *It is indicated to perform a brain MRI with gadolinium in case of dermatological suspicion of SWS, preferably after the age of 12 months. Moreover, brain MRI with gadolinium is indicated in all subjects with onset of neurological signs/symptoms with a negative brain MRI performed before the age of 12 months or who never had a neuroimaging. If the patient is asymptomatic and has performed a negative MRI before the age of 12 months, it is preferable to wait the age of cooperation (usually after 6 years) to repeat neuroimaging, thus avoiding sedation.*  *Whenever possible, in case of high suspicion, a first feed-and-wrap approach (sleep or oral midazolam under monitoring of vital parameters) within 4 weeks of life can be suggested, preferably with a 3 Tesla scanner, avoiding gadolinium injection, for the detection of intracranial involvement, but taking into account the possibility of false negatives.* | 91% (30/33) of experts rated this recommendation ≥4 on a Likert scale at first round |
| 1. **Is brain computerized tomography (CT) indicated in patients with dermatological suspicion of SWS?**   *Brain CT is not indicated since it has poor sensitivity, moreover it exposes the patient to a risk associated with ionizing radiation.* | 94% (31/33) of experts rated this recommendation ≥4 on a Likert scale at first round |
| 1. **Is neuroradiological follow-up indicated in patients with confirmed SWS?**   *Control brain MRIs in patients with confirmed SWS are indicated only in case of unexpected worsening of neurological signs/symptoms, with the aim to evaluate the progression of brain involvement (calcifications, atrophy). Control MRIs during acute events such as status epilepticus, migraine or stroke-like episodes can show elements useful to understand the pathogenesis of the event, but do not change the clinical management.* | 94% (31/33) of experts rated this recommendation ≥3 on a Likert scale at first round |
| 1. **Is presymptomatic treatment with antiepileptic drugs indicated in children with SWS?**   *Early presymptomatic treatment with antiepileptic medications may be offered to patients with extensive unilateral or bilateral brain involvement, at higher risk for early-onset epilepsy, after having explained to families their empirical use and possible adverse events.*  *It is recommended to inform and educate families to the use of rescue therapies in case of prolonged seizures and/or cluster of seizures, associated to appropriate treatment of fever and dehydration.* | 82% (27/33) of experts rated this recommendation ≥3 on a Likert scale at second round |
| 1. **What are the most appropriate anti-seizure medications (ASMs) in patients with SWS and epilepsy?**   *Antifocal ASMs such as oxcarbazepine, carbamazepine and levetiracetam are considered the first-line therapy. When ineffective, or in patients not eligible for surgery, alternative or adjunctive therapies are lacosamide, lamotrigine, fenobarbital, cannabidiol, topiramate, clobazam, and ketogenic diet. For patients with frequent headaches a shift to valproic acid or lamotrigine should be considered. Home-administered benzodiazepines (buccal/intranasal midazolam and/or rectal diazepam) are first-line agents for acute and prolonged seizures and clustered seizure management. Specialist decisions on pharmacological treatment should be personalized, and consider seizure types, patient age, and possible comorbidities.* | 82% (27/33) of experts rated this recommendation ≥3 on a Likert scale at first round |
| 1. **What are the most appropriate drugs in case of headache or migraine?**   *At present, there are no guidelines on acute and preventative management of headache. In most countries, sleep, hydratation, ibuprofen, paracetamol and antiemetics are used. It is important to consider that in younger patients with cluster of seizures associated to irritability/neurological signs, analgesic treatment combined to a short-term benzodiazepine pulse should give relief and shorten the acute phase. Data from the literature confirm that triptans are safe and frequently effective. The most used drugs for prophylaxis are anti-seizure medications (valproate, lamotrigine, topiramate or gabapentin) and flunarizine.* | 82% (27/33) of experts rated this recommendation ≥3 on a Likert scale at first round |
| 1. **Is acetylsalicylic acid (ASA) indicated?**   *Despite the absence of evidences, low-dose ASA (3 to 5 mg/kg/day to a maximum of 100 mg/day) should be offered early in the disease course, after the appearance of neurological signs/symptoms, in patients with extensive brain involvement and/or cluster of seizures associated to subsequent neurological worsening.* | 79% (26/33) of experts rated this recommendation ≥4 on a Likert scale at second round |
| 1. **When is a specialist evaluation for the surgical treatment of epilepsy indicated?**   *Surgical indication should be reasonably considered in drug-resistant patients, mostly in subjects with early onset of seizures and pre-existing developmental impairment and/or neurological deficit. We cannot suggest any specific type of surgical approach since every centre for epilepsy surgery has its proper techniques.*  *Type of surgery (hemispherotomy, sublobar/lobar resections, multilobar disconnections) depends on the extension of the epileptogenic area and its relation with functional areas, and has to be always evaluated in a third level centre for epilepsy surgery.*  *Early surgery should have a favourable role on cognitive and motor outcome. In selected cases with bilateral involvement, hemispherotomy on the more active hemisphere should be considered, with palliative intent.* | 79% (26/33) of experts rated this recommendation ≥4 on a Likert scale at second round |
| 1. **Do children with SWS need psychomotor/psychodiagnostic screening?**   *Considering the high prevalence of neurocognitive and behavioural comorbidities in children with SWS, a standardized screening of their evolutionary trajectory can lead to early rehabilitative treatment. A developmental/neurocognitive evaluation is recommended at diagnosis, at seizure onset, in case of suspected neurodevelopmental delay and appearance of neurocognitive/behavioural abnormalities.*  *The primary care paediatrician should regularly supervise children’s development during follow-up. In case of suspected developmental delay, it is recommended to refer the patient for neurodevelopmental or neurocognitive evaluation using standardized scales (e.g. Bayley, Griffiths) with the aim to start rehabilitation therapies and an individualized educative plan.* | 85% (28/33) of experts rated this recommendation ≥4 on a Likert scale at second round |
| 1. **What is the psychological impact of SWS on patients and their parents and how it should be managed?**   *Psychological implications of SWS are widely underexplored and underestimated both in early ages (neurodevelopmental disorders, emotional-behavioural issues, and learning difficulties) and in adulthood (mood disorders, low self-esteem, emotional disorders, social isolation).*  *In case of clinical doubt of discomfort or psychological issues, it is recommended to refer the patient for a psychodiagnostic evaluation. This assessment can establish the need of psychotherapy or welfare/social support, offer parents’ counselling, and evaluate the aesthetic impact and the stigma perceived by patients and/or their families.* | 85% (28/33) of experts rated this recommendation ≥4 on a Likert scale at second round |
| 1. **Ophthalmological recommendations** |  |
| 1. **In infants with a CM suggestive of SWS, what parameters other than intraocular pressure (IOP) are relevant for the diagnosis of glaucoma?**   *The ocular parameters relevant for the diagnosis of glaucoma are, in addition to the IOP, the variation of the corneal diameter, axial length and cup-to-disc ratio. Blepharospasm, tearing and photophobia are also suspicious symptoms.* | 88% (29/33) of experts rated this recommendation ≥3 on a Likert scale at first round |
| 1. **Can fundus examination reveal retinal changes? When can the use of diagnostic retinal imaging be justified and with which tools?**   *Fundus examination shows retinal alterations related to choroidal vascular malformations in many cases. Diagnostic imaging should be performed to identify and monitor specific ocular anomalies. Optical Coherence Tomography (OCT) is used to define the extent of the lesion and its location, and angiography in suspected exudation. Ultrasound is used to define the reflectivity of the lesion; in children or in non-cooperative patients, it can be performed under anesthesia. Fundus photography with possible angiography is employed in children or non-cooperative patients, in the suspicion of exudation.* | 91% (30/33) of experts rated this recommendation ≥4 on a Likert scale at first round |
| 1. **Is it advisable to maintain ophthalmological follow-up in patients with SWS life-long?**   *Since the risk of developing glaucoma is present throughout life, monitoring must be regular and continuous. It is advisable to carry out at least annual outpatient check-ups from birth, in the absence of complications. The follow-up will then be personalized, for example in case of amblyopia, glaucoma or retinal vascular malformation. Tonometry will be performed under anesthesia until the patient is cooperative and can undergo outpatient check-up.* | 91% (30/33) of experts rated this recommendation ≥3 on a Likert scale at first round |
| 1. **Does pharmacological therapy play a role in the management of glaucoma in patients with SWS?**   *Infantile glaucoma u*sually *requires surgical therapy; medical therapy is used only in the initial phase of the disease, pending intervention. In adult glaucoma, pharmacological therapy can be used, depending on the degree of ocular hypertension. The international guidelines for the management of glaucoma apply.* | 85% (28/33) of experts rated this recommendation ≥3 on a Likert scale at first round |
| **5. What are the indications for surgery and which type of surgery is most suitable for the treatment of glaucoma in SWS?**  *Surgery is indicated when IOP is not controlled despite appropriate and maximal pharmacological therapy, due to intolerance or allergy to hypotonic medications, or therapy non-compliance. Surgery involves penetrating filtering procedures for glaucoma. Retro equatorial drainage systems are used as a last choice.* | 82% (27/33) of experts rated this recommendation ≥3 on a Likert scale at first round |
| **6. When is laser treatment indicated for the treatment of retinal vascular malformations?**  *Laser treatment of choroidal vascular malformations should be performed when there is a risk of complications such as subretinal hemorrhage, serous retinal detachment, cystoid macular edema and neuroepithelial detachment.* | 88% (29/33) of experts rated this recommendation ≥3 on a Likert scale at first round |
| 1. **Dentistry recommendations** |  |
| 1. **When should the patient with SWS be referred to the dentist and how should follow-up be planned?**   *The first dental assessment should be performed as soon as the diagnosis of SWS has been made, in order to promptly evaluate possible mucosal vascular lesions, dental alterations in terms of size and/or timing of tooth replacement, and skeletal asymmetries.*  *If there are no lesions in the oral mucosa, follow-up should be every six months.*  *If lesions are present, follow-up should be every four months to assess periodontal health status, tendency to spontaneous bleeding, degree of gingival hypertrophy, and any skeletal and functional consequences.* | 82% (27/33) of experts rated this recommendation ≥4 on a Likert scale at second round |
| 1. **What are the age-related dental assessments of SWS patients?**   *- 0 to 3-year-old: investigate the presence of any vascular lesion in the oral and perioral soft tissues, assess facial symmetry and the status of eruption of the deciduous series. Establish a correct oral prevention and prophylaxis protocol by age, according to national guidelines.*  *- 3 to 6-year-old: carry out the evaluations listed above and monitor the first phase of tooth replacement and any jawbone asymmetry.*  *- 6 to 12-year-old: carry out the assessments listed above and monitor the transition from mixed to permanent dentition to early detect any abnormalities. Perform an initial panoramic radiography. Assess periodontal health status. Investigate any malocclusion related to a skeletal asymmetry ipsilateral to the CM and lingual posture.*  *- >12-year-old: monitor vascular lesions and perform orthodontic evaluation of dental occlusion. If needed, joint assessment with the maxillofacial surgeon to discuss indications to pre-surgical orthodontics or skeletal compensation. Assess periodontal health status.* | 79% (26/33) of experts rated this recommendation ≥4 on a Likert scale at second round |
| 1. **Can laser treatment be effective in the management of vascular lesions of the oral cavity in SWS patients?**   *Laser treatment appears to be an effective therapy for the management of vascular lesions of the oral mucosa. It allows gingivectomy of the hypertrophic component and non-surgical periodontal therapy through decontamination of the gingival sulcus. The choice of the type of laser depends on the treatment to be performed.* | 85% (28/33) of experts rated this recommendation ≥3 on a Likert scale at second round |
| 1. **Plastic surgery recommendations** |  |
| 1. **When is orthognathic surgery indicated in patients with SWS?**   *Based on recent literature, patients affected by SWS who underwent orthognathic surgery show satisfactory results, comparable to standard patients. Patients with SWS, who have adequate neuro-cognitive development and therefore can ask for treatment, can undergo orthognathic surgical treatments for the correction of dento-skeletal malocclusions.* | 79% (26/33) of experts rated ≥4 this recommendation on a Likert scale at second round |
| 1. **What is the role of plastic surgery in patients with SWS and CM associated with tissue hypertrophy?**   *Surgery in patients with SWS is nowadays believed to be safe and effective with only a modest increase in bleeding risk. Indications for surgery include debulking of CM areas with significant tissue hypertrophy (especially the lip), removal of major or minor nodules (when the scarring will be more acceptable for the patient than the lesion), or recurrent bleeding.* | 79% (26/33) of experts rated ≥4 this recommendation on a Likert scale at second round |
| 1. **Primary care paediatrician recommendation** |  |
| 1. **Which is the role of the primary care paediatrician (PCP) in the care of children with SWS?**   *If the PCP suspects SWS, he/she should refer the child to a Reference Centre. For children with confirmed SWS, the PCP should follow the patient and family in accordance with the Reference Centre. The role of PCP is fundamental to ensure the best QoL and appropriate patient management by caregivers. The PCP should monitor child growth and neurodevelopment, identify signs/symptoms of suspect hypothyroidism, and/or growth hormone deficiency.* | 91% (30/33) of experts rated this recommendation ≥4 on a Likert scale at first round |
